# Alterations in the health of hibernating bats under pathogen pressure

**DOI:** 10.1038/s41598-018-24461-5

**Published:** 2018-04-17

**Authors:** Hana Bandouchova, Tomáš Bartonička, Hana Berkova, Jiri Brichta, Tomasz Kokurewicz, Veronika Kovacova, Petr Linhart, Vladimir Piacek, Jiri Pikula, Alexandra Zahradníková, Jan Zukal

**Affiliations:** 1 Department of Ecology and Diseases of Game, Fish and Bees, University of Veterinary and Pharmaceutical Sciences Brno, Brno, Czech Republic; 2 Department of Botany and Zoology, Masaryk University, Brno, Czech Republic; 3 Institute of Vertebrate Biology, Czech Academy of Sciences, Brno, Czech Republic; 4 Institute of Biology, Department of Vertebrate Ecology and Palaeontology, Wrocław University of Environmental and Life Sciences, Wrocław, Poland; 5 CEITEC - Central European Institute of Technology, University of Veterinary and Pharmaceutical Sciences Brno, Brno, Czech Republic; 6 Department of Muscle Cell Research, Institute of Molecular Physiology and Genetics, Centre of Biosciences, Slovak Academy of Sciences, Bratislava, Slovakia

## Abstract

In underground hibernacula temperate northern hemisphere bats are exposed to *Pseudogymnoascus destructans*, the fungal agent of white-nose syndrome. While pathological and epidemiological data suggest that Palearctic bats tolerate this infection, we lack knowledge about bat health under pathogen pressure. Here we report blood profiles, along with body mass index (BMI), infection intensity and hibernation temperature, in greater mouse-eared bats (*Myotis myotis*). We sampled three European hibernacula that differ in geomorphology and microclimatic conditions. Skin lesion counts differed between contralateral wings of a bat, suggesting variable exposure to the fungus. Analysis of blood parameters suggests a threshold of ca. 300 skin lesions on both wings, combined with poor hibernation conditions, may distinguish healthy bats from those with homeostatic disruption. Physiological effects manifested as mild metabolic acidosis, decreased glucose and peripheral blood eosinophilia which were strongly locality-dependent. Hibernating bats displaying blood homeostasis disruption had 2 °C lower body surface temperatures. A shallow BMI loss slope with increasing pathogen load suggested a high degree of infection tolerance. European greater mouse-eared bats generally survive *P. destructans* invasion, despite some health deterioration at higher infection intensities (dependant on hibernation conditions). Conservation measures should minimise additional stressors to conserve constrained body reserves of bats during hibernation.

## Introduction

Life history theory suggests that organisms optimise their defences against pathogens by differential allocation of resources to support different physiological functions^1–4^. There is a trade-off mechanism applied to modulate investment into individual life history components^5,6^. Homeostasis and survival of hosts challenged with exposure to a pathogenic agent could be considered a physiological measure of health^2^. Host physiological status under pathogen pressure will be impacted by standard components of the ‘disease triangle’, i.e., host susceptibility, virulence of the infectious agent and environmental determinants. In general, hibernation, a slow life history strategy, is linked to higher survival rates^7^. Successful hibernation of mammals is constrained by their energy reserves, suitable microhabitat availability^8^ and thermoregulatory behaviour^9^. Additional stressors may deplete the animal’s resources, resulting in adverse consequences.

Skin, the largest organ of the body, acts as a barrier between the animal and its environment while providing multiple anatomic and physiological functions. A bat’s membranes are essential for flight, increasing the ratio of body mass to body surface. Moreover, naked flight membranes have a surface area eight times greater than that of fur-coated skin. This increases the area of potential exposure to dermatopathogens. In bats, healthy skin is essential for maintaining physiological homeostasis^10^.

In the northern temperate zone, hibernating bats are exposed to a non-systemic fungal infection that mainly affects the areas of skin without fur. Over the last decade, the fungus *Pseudogymnoascus destructans* has caused a devastating decline in North American bat populations^11–16^. During this time, there have been only sporadic cases of mortality in Eurasia^17–21^. In contrast to standard cutaneous dermatomycoses, the so-called white-nose syndrome (WNS) fungus invades living layers of skin^10,22,23^.

Despite considerable advances in our understanding of molecular pathogenesis and factors affecting the virulence of *P. destructans* infection^24–27^, the fundamental pathophysiological mechanisms of mortality associated with WNS remain unconfirmed^10,28,29^. Adverse effects increase with the extent of wing membrane pathology. While early stages of skin infection induce a two-fold increase in fat energy utilisation^29^, late-stage infected bats have altered torpor-arousal cycles, abnormal hibernation behaviour as well as emaciation and increased mortality^30^. Several studies of clinical blood parameters (e.g. electrolytes, acid-base balance, hydration status, haematology) in little brown bats, *Myotis lucifugus*, reveal that WNS disrupts blood homeostasis^29,31,32^. Interestingly, European *P. destructans* isolates are virulent and produce WNS in this North American bat species^33^. Infected bats have histopathology identical to skin lesions in Palearctic bat species^18–22^. *P. destructans* occurred in Europe before the outbreak of the Nearctic epidemic^34,35^. This, together with phylogenetic studies^36,37^, indicates that North American species of bats might be naïve hosts to a fungal pathogen originating in the Palearctic region. Intercontinental and interspecies comparisons may provide greater insights into variation of host responses to fungal infection.

Two defence mechanisms can evolve from host-pathogen interactions: resistance and tolerance^38–40^. Resistance protects the host by reducing the pathogen burden. As a consequence, prevalence of the agent in the host population decreases. In comparison, tolerant hosts limit the damage caused by the pathogen and remain healthy without mounting sterilising immunity^41^, though prevalence remains high or even increases within the susceptible population. Host cost/benefit trade-offs from its response to infection should favour tolerance when disease severity allows survival and host adaptation^42^. As European bats infected with *P. destructans* display no population-level effects, they are thought to tolerate infection, despite high fungal loads and almost 100% prevalence^17,20,21^. Host tolerance and/or disease resistance can be measured as a regression slope between health and pathogen load^38,42^.

Here, we report on host-pathogen interactions in the greater mouse-eared bat (*Myotis myotis*), the European species showing highest skin infection intensity, based on blood parameters. We hypothesise that hibernating European bats are unable to maintain blood parameters within the normal physiological ranges found in healthy bats when exposed to the WNS fungal agent, and that blood homeostasis disruption could be related to infection intensity and hibernation temperature. We predict 1) a skin lesion threshold distinguishing healthy and diseased bats, 2) blood acidosis and a decrease of blood glucose in bats with high *P. destructans* infection intensity, and 3) a lower body mass index (BMI) in bats with blood homeostasis disruption. We also predict a reduced rate of BMI loss with increasing infection intensity, indicative of disease tolerance. An improved understanding of how hibernating bats optimise their health to survive pathogenic pressure will have positive ramifications for wildlife and conservation medicine.

## Methods

### Ethics statement

Each bat was handled in such a way as to minimise sampling distress and was released at the hibernaculum one hour after capture. Fieldwork and bat sampling was performed in accordance with Czech Law No. 114/1992 on Nature and Landscape Protection, based on permits 1662/MK/2012S/00775/MK/2012, 866/JS/2012 and 00356/KK/2008/AOPK issued by the Agency for Nature Conservation and Landscape Protection of the Czech Republic. Experimental procedures were approved by the Ethical Committee of the Czech Academy of Sciences (No. 169/2011). Sampling at the Nietoperek Natura 2000 site (Poland) was approved by the II Local Ethical Commission in Wrocław (No. 45/2015) and the Regional Nature Conservancy Management in Gorzów Wielkopolski (WPN-I-6205.10.2015.AI and WPN-I-6205.20.2016.AI). The authors were authorised to handle free-living bats under Czech Certificate of Competency No. CZ01341 (§17, Act No. 246/1992) and Polish Certificate of Competency in Experimental Procedures on Animals (Polish Laboratory Animal Science Association, Certificate No. 2413/2015).

### Hibernacula studied

Seventy-nine bats were sampled at three important European hibernacula, the Nietoperek bunker (NIE; Poland), the Šimon and Juda mines (SJM; Czech Republic) and the Sloupsko-Šošůvské caves (SSC; Czech Republic), during the late hibernation period in 2015. Control sampling was undertaken at the Nietoperek bunker during March 2016 to evaluate infection dynamics. All three localities differ in geomorphology and microclimate conditions. No mass mortalities have been reported from any of the sites^43–46^ and numbers of hibernating *M. myotis* have remained stable, or have increased slightly over recent years.

The Nietoperek bat reserve lies in the underground corridors of an abandoned German military fortification from the central sector of the Międzyrzecz Fortified Front in western Poland (52°25′N, 15°32′E). The aboveground bunkers are connected by 3–4.5 m high and 2.5–4 m wide underground railway corridors. Sites preferred by hibernating *M. myotis* have a median temperature of 8.7 °C (min-max 6.1–9.9 °C), 100% relative humidity (min-max 77.5–100.0%) and 9 g/m^3^ absolute humidity (min-max 6–9 g/m^3^)^46^.

The Šimon and Juda mines comprise two gallery systems, the entrances of which open into a 10 m deep iron ore quarry. The mines were closed in 1870 and most galleries were flooded after World War I. While the galleries were drained between 1956 and 1957, no more mining took place. The lower gallery system has four horizontal storeys, each 2–2.5 m high, the upper system comprising an irregular labyrinth of galleries and chambers. Differences in geomorphology mean that each system has a different microclimate, the lower being colder, with temperatures rarely exceeding 5 °C (relative humidity close to 100%; absolute humidity 7–8 g/m^3^) and the upper having temperatures around 7 °C in most parts, though dropping close to the main entrance^47^.

The Sloupsko-Šošůvské caves comprise a natural karst system with 7 km of chasms, domes and corridors. The 8 m high and 20 m wide main entrance is located in the northern part of the cave. Due to their complicated geomorphology, microclimatic conditions vary widely. Hibernating bats mainly use those parts close to the main entrance (e.g. the Nicová and Eliška cave^45^) with mean annual temperatures fluctuating between 5.5 and 7.5 °C and an absolute humidity of 7–8 g/m^3^ ^48^.

### Measurements of bat health

The body surface temperature of each hibernating bat was measured using a Ryatek contactless laser thermometer (Total Temperature Instrumentation Inc.) prior to its removal from the hibernaculum wall. Each bat was then sexed and its age estimated based on epiphyseal ossification of the thoracic limb fingers and tooth abrasion^49^. Callipers were used to measure forearm length and body mass was determined using a portable top-loading balance. BMI was calculated as body mass (g) divided by left forearm length (mm)^50^.

After a re-warming period of 60 minutes, the skin was disinfected with alcohol and a blood sample (100 µl) taken from the uropatagial vessel using a heparinised tube^51^. An i-STAT portable clinical analyser (EC8+ diagnostic cartridge, Abaxis, Union City, CA, USA) was used to measure sodium (Na, mmol/L), potassium (K, mmol/L), chloride (Cl, mmol/L), total dissolved carbon dioxide (tCO_2_, mmol/L), blood urea nitrogen (BUN, mmol/L), glucose (GLU, mmol/L), haematocrit (Hct, L/L), pH, partial dissolved carbon dioxide (pCO_2_, kPa), bicarbonate (HCO_3_, mmol/L), base excess (BE, mmol/L), anion gap (AnGap, mmol/L) and haemoglobin (Hb, g/L).

A subsample was used to prepare a blood smear, which was then treated with Romanowsky stain. Differential white blood cell counts were determined by counting 100 leukocytes under oil immersion magnification and calculating the relative number of lymphocytes, monocytes, neutrophils, basophils and eosinophils.

### Measurement of infection intensity

Immediately following capture, the surface of the left wing was swabbed (FLOQ Swabs, Copan Flock Technologies srl, Brescia, Italy) in a standardised manner to collect fungal biomass. Fungal load was calculated using quantitative polymerase chain reaction (qPCR) and the QIAamp DNA Mini Kit (Qiagen, Halden, Germany) was used to isolate fungal DNA from the wing swabs. A dual-probe TaqMan (Life Technologies, Foster City, CA, USA) was used to quantify *P. destructans* DNA (ng per left wing area; triplicate samples) using a previously described protocol employing positive and negative controls and a dilution series calibration curve from a positive control^19,21,52,53^. Suspected fungal growths from other parts of the body (e.g. ears, muzzle) were collected for laboratory culture examination^12^. Skin lesions were enumerated by photographing both wings over a 368 nm ultra-violet (UV) lamp^19,21,54^. A 4 mm punch biopsy, centred over the lesion, was collected from each bat to confirm *P. destructans* infection on histopathology^22,23^.

### Statistical analysis

Normality of variable distribution was tested using the Shapiro-Wilk test. Non-normally distributed variables were log transformed and rechecked. All parameters were normally distributed after transformation, with the exception of body surface temperature (W = 0.936, p = 0.005), haematocrit (W = 0.896, p < 0.001), haemoglobin (W = 0.896, p < 0.001) and percentage of eosinophils, monophils and basophils (Shapiro-Wilk tests, p < 0.001). In these cases, statistical analysis was conducted using non-parametric tests, i.e. the Kruskal-Wallis test, the Mann-Whitney U test and Spearman’s correlation. The slopes and intercepts of linear regressions were compared using the Student’s t-test.

Haematological parameters did not differ between age classes (adult vs. sub-adult) or sexes (ANOVA and t-test); hence, the data were pooled for subsequent analyses. We scored the level of wing damage based on the total number of UV-fluorescing skin lesions on both wings as: 1 = 0 to 50 lesions, 2 = 51 to 250 lesions, 3 = 251 to 500 lesions, 4 = 501 to 1000 lesions and 5 = more than 1000 lesions. Effect of locality and wing-lesion score on blood parameters was tested using general linear mixed models (GLMM) with locality (hibernaculum) set as a random effect. As blood parameters were highly inter-correlated, we used principal component analysis (PCA) to evaluate inter-individual differences in distribution along axes linked with severity of skin infection, i.e. number of UV-fluorescing skin lesions.

Data on fungal load, number of UV-fluorescing skin lesions and blood parameters from Nietoperek (Poland), the Šimon and Juda mines (Czech Republic) and the Sloupsko-šošůvské caves (Czech Republic) are presented in Supplementary Table S1.

## Results

### Relationship between number of skin lesions, body surface temperature, BMI and *P. destructans* load

**Figure 1:**
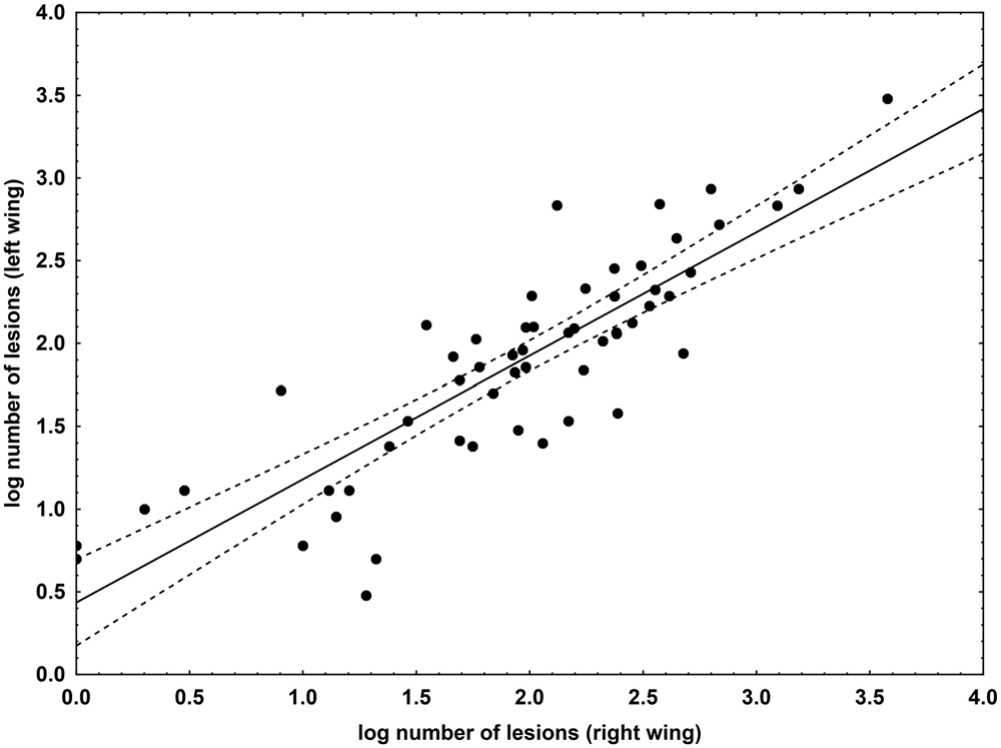
Relationship between the number of skin lesions produced by *P. destructans* on the left and right wing of each bat. Displayed as a scatter plot of log-transformed data, it indicates a positive correlation between the left and right wing lesion counts. Points lying outside the 95% confidence intervals of the regression line show that one wing had more UV-fluorescing lesions than the contralateral wing in a given bat.

**Figure 2:**
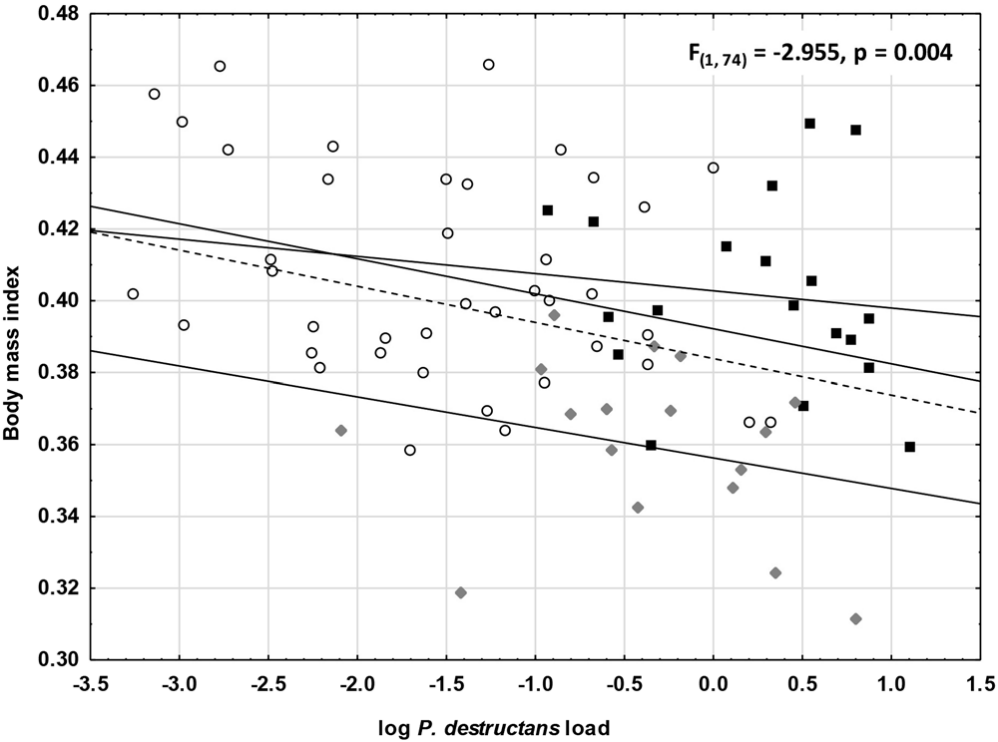
Relationship between BMI and *P. destructans* load for bats from different localities. *F*- and *p*-values are given to demonstrate the effect of *P. destructans* load on bat BMI in the pooled material. Dots = Nietoperek (NIE); squares = Šimon and Juda mine (SJM); diamonds = Sloupsko-šošůvské cave (SSC); dashed line = regression line for pooled dataset.

**Figure 3:**
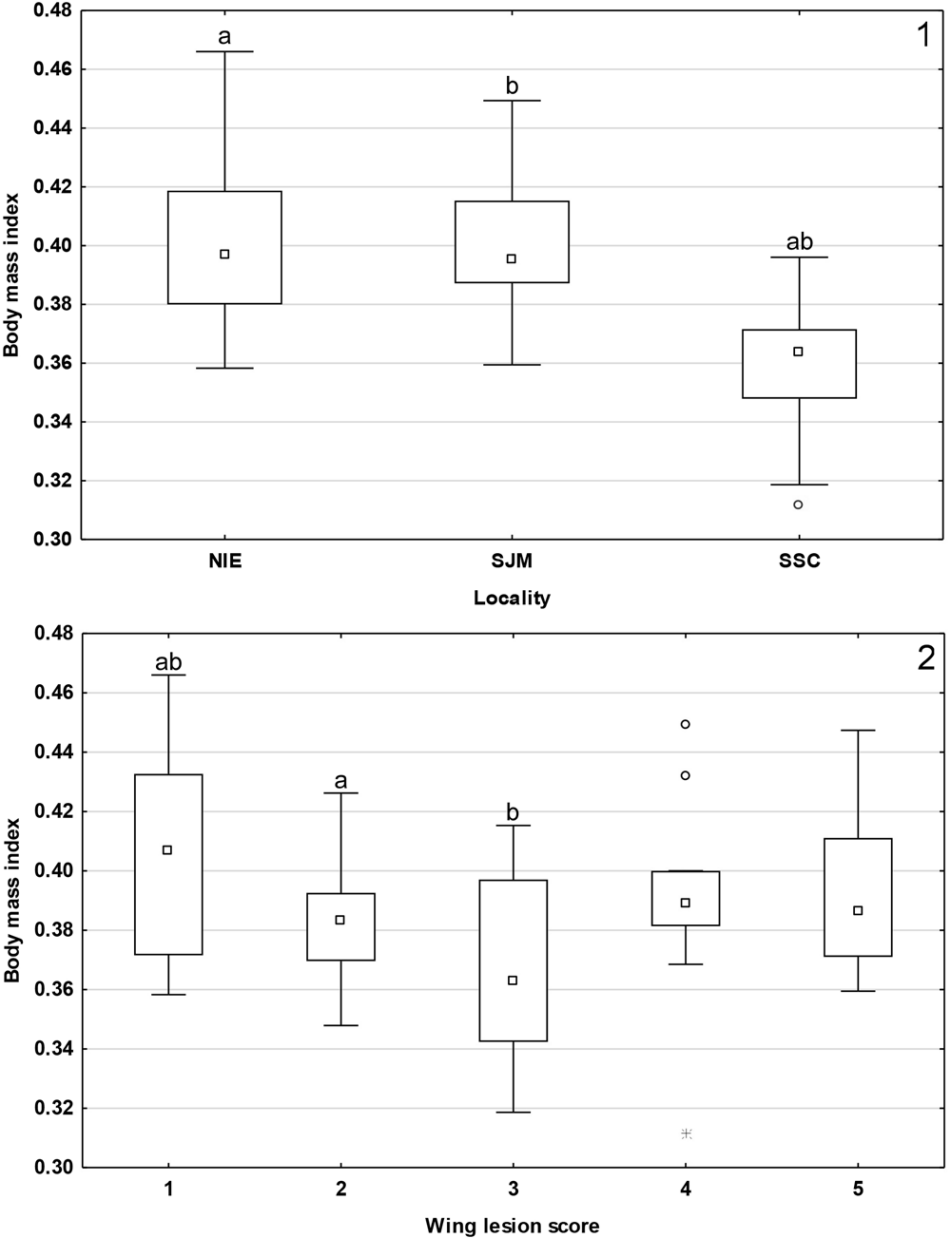
Median BMI of bats from (1) different localities, and (2) with different wing-lesion scores. Midpoint = median, box = inter-quartile range, whiskers = non-outlier range, dots = outliers, stars = extremes. Groups marked with the same letter differ significantly.

The number of skin lesions fluctuated between 0 and 3782. One wing for a given individual always had significantly more lesions (Wilcoxon Matched Pairs Test; Z = 2.497, p = 0.013; Fig. 1), though the number of lesions on each wing was correlated (Spearman rank order correlation r_s_ = 0.857, p < 0.05). Consequently, we used the sum of UV-fluorescing lesions from both wings as it more precisely expressed total skin infection severity. Number of lesions was positively correlated with *P. destructans* load and negatively with body surface temperature (Table 1). The decline in BMI with higher *P. destructans* load was the same at all localities, though the regression curve for the Sloupsko-Šošůvské caves did not differ significantly at its lower intercept (SSC vs. NIE t = −0.303, DF = 53, p = 0.382; SSC vs. SJM t = −0.141, DF = 32, p = 0.445) due to the lowered BMI of hibernating bats (Figs 2 and 3). When material from all localities and both years of sampling was pooled, the decline in BMI with increasing *P. destructans* load and number of skin lesions became statistically significant (*P. destructans* load F_(74)_ = −2.955, p = 0.004; UV-fluorescing skin lesions F_(75)_ = −2.461, p = 0.016).

**Table 1:** Spearman rank order correlation between body parameters and infection parameters.

Variable	**Number of skin lesions**	**Body surface temperature**	**Body mass index**
Body surface temperature	**−0.54**		
Body mass index	−0.15	−0.07	
*P. destructans* load	**0.69**	**−0.61**	−0.12

Figures in bold are significantly different at α < 0.05.

**Figure 4:**
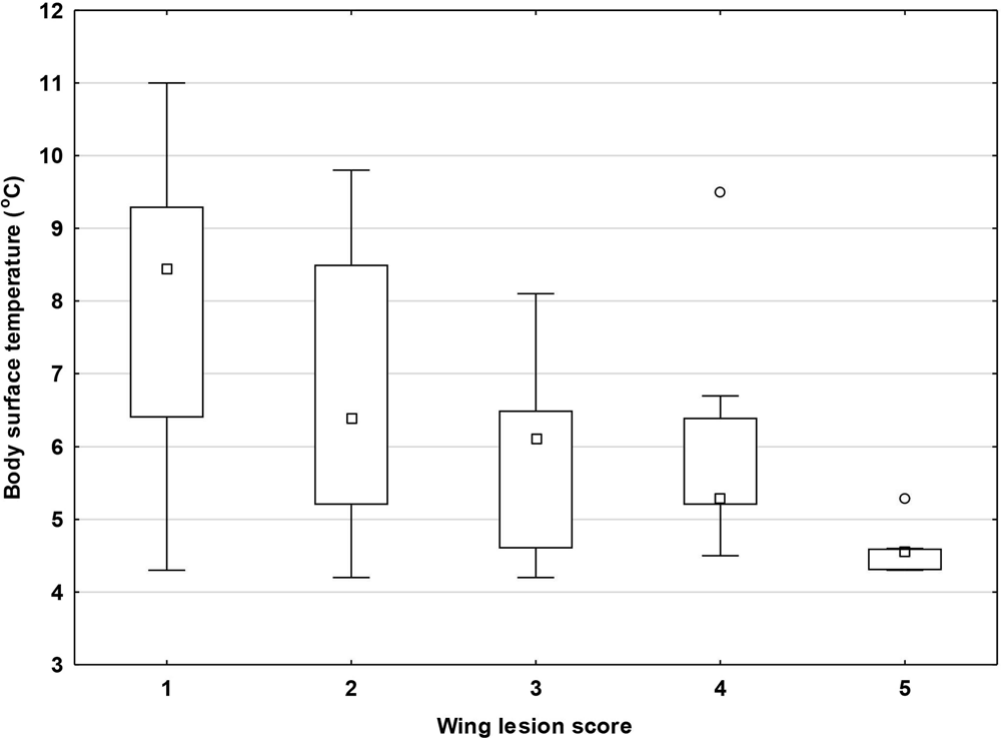
Body surface temperature of hibernating bats with different wing-lesion scores. Midpoint = median, box = inter-quartile range, whiskers = non-outlier range, dots = outliers. Body surface temperature was significantly different between wing-lesion scores (Kruskal-Wallis test: H_4,57_ = 16.773; p = 0.002).

GLMM confirmed both the differences in *P. destructans* load (F = 8.106, p < 0.001) and BMI (F = 7.785, p < 0.001). While post-hoc univariate tests indicated locality as the main effect for *P. destructans* load; BMI was significantly influenced by both locality and wing-lesion score (Fig. 3). Highest BMI scores were recorded at lowest infection severity (score 1). Kruskal-Wallis tests confirmed a difference in body surface temperature for both locality (H_(2, N = 57)_ = 49.826, p < 0.001) and wing-lesion score (H_(4, N = 57)_ = 16.773, p = 0.002), hibernating bats with lowest body surface temperatures showing increased infection severity (Fig. 4).

### Relationship of skin infection level to blood chemistry and haematology profile

**Figure 5:**
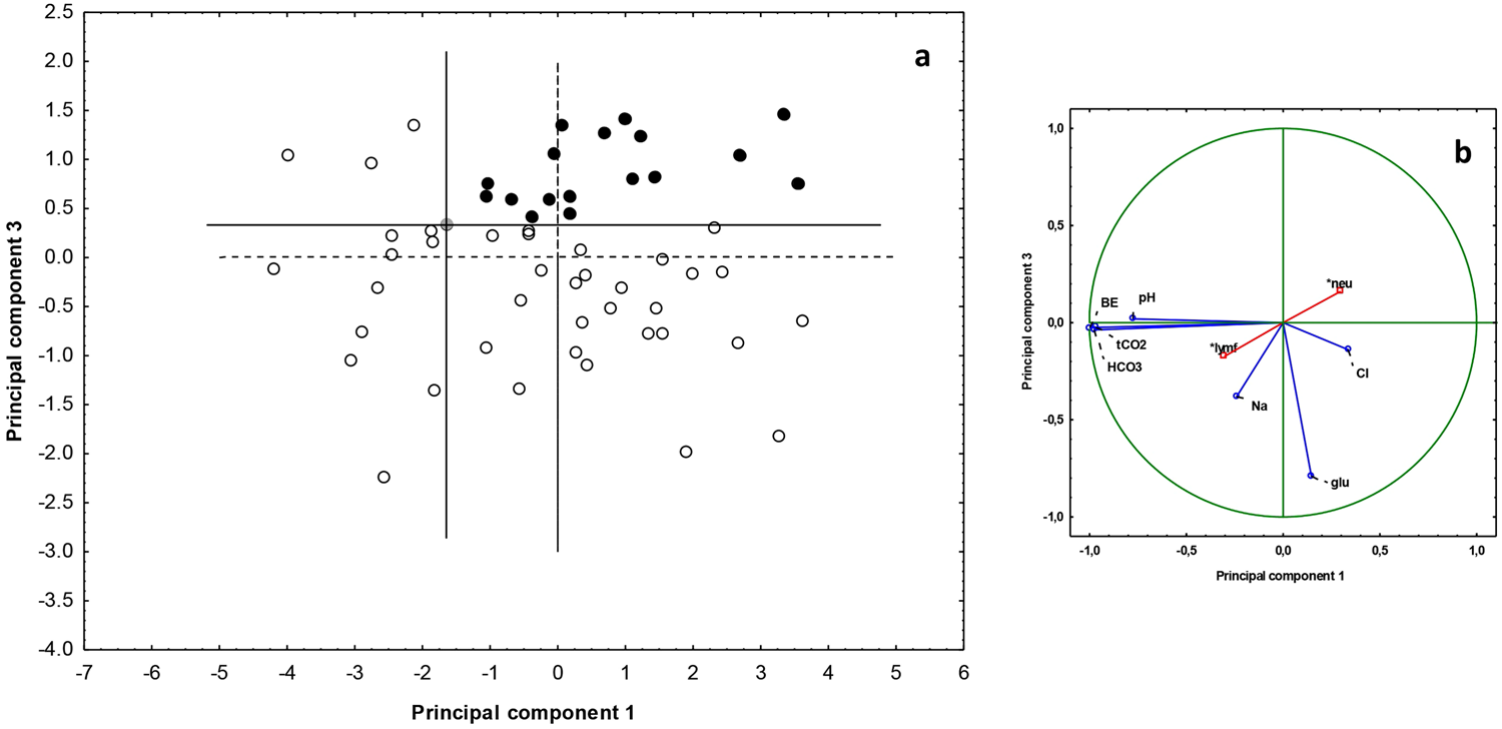
(**a**) Bat dispersion and (**b**) projection of variables in blood parameter space based on PCA. The position of bats with no UV-fluorescing skin lesions and highest principal component values (grey dot) was used to define the midpoint of new principal component axes. Black dots = diseased, open dots = healthy; supplementary factors are marked by a star. Abbreviations: BE = base excess, glu = glucose, lymf = lymphocytes, neu = neutrophils.

**Figure 6:**
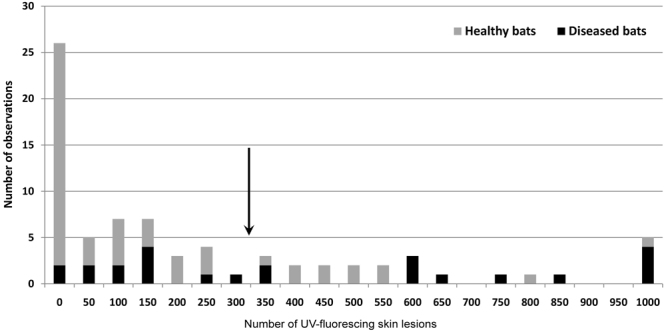
Frequency of skin lesions. Bats were identified as either healthy (n = 52) or with homeostasis disrupted by *P. destructans* skin infection (n = 24) using PCA. The arrow shows the expected threshold in number of UV-fluorescing skin lesions (328.5) for manifestation of skin infection through disruption of blood parameters.

Nine blood parameters were significantly affected by locality and wing-lesion score (Tables 2 and 3), the GLMM model explaining between 14.3 and 37.7% of variability. We used seven continuous blood parameters (excluding percentage of neutrophils and lymphocytes) for PCA analysis of samples from 2015, the first three components of which explained 92.5% of total variability. Acid-base variables (tCO_2_, pH, HCO_3_ and BE) displayed a strong negative correlation with the first component, electrolytes (Na and Cl) correlated positively with the second component and glucose negatively with the third component. The space defined by the first and third components provided the best separation between individuals (Fig. 5). Highest principal component values were observed in a single healthy individual (without UV-fluorescing skin lesions), its position subsequently being considered a new midpoint for the principal component axes. All individuals (n = 18) located in the upper right space were diagnosed with homeostasis disruption associated with skin infection, the three worst cases (top right position in Fig. 5) displaying infection intensities of 2394, 876 and 1490 lesions. As all bats from Nietoperek proved healthy, we took control samples during winter 2016 and repeated the PCA analysis with a larger sample size. While there was no difference in BMI and skin infection intensity in 2015 and 2016 (T-test; t = −1.598, p = 0.118, and t = 1.154, p = 0.256); *P. destructans* load was higher in 2015 than 2016 (T-test; t = 3.176, p = 0.003). PCA added seven new cases to those diagnosed with homeostasis disruption, including two from Nietoperek. Average number of skin lesions, *P. destructans* load, BMI and body surface temperature differed significantly between healthy and diseased bats (Table 4). We defined a theoretical breaking point (skin lesion threshold) for the manifestation of skin infection in disruption of blood parameters, based on the median between the 95% confidence intervals of the two groups (i.e. 328.5 skin lesions; Fig. 6). Six blood parameters differed between the groups defined by PCA; however, only glucose, skin lesion number, log *P. destructans* load and body surface temperature differed between the groups defined by the skin lesion threshold (Table 4).

**Table 2:** Summary statistics for general linear models of blood parameters, with wing-lesion score as a fixed factor and locality as a random factor.

Dependent Variable	Adjusted R^2^	df Model	df Residual	F	p
Na	**0.143**	**6**	**52**	**2.609**	**0.028**
K	0.059	6	52	1.602	0.165
Cl	**0.158**	**6**	**50**	**2.749**	**0.022**
tCO_2_	**0.342**	**6**	**51**	**5.946**	**<0.001**
Urea	−0.019	6	51	0.823	0.558
Glucose	**0.152**	**6**	**51**	**2.705**	**0.023**
pH	**0.259**	**6**	**51**	**4.314**	**0.001**
pCO_2_	0.072	6	51	1.739	0.131
HCO_3_	**0.348**	**6**	**51**	**6.061**	**<0.001**
Base excess	**0.377**	**6**	**51**	**6.746**	**<0.001**
Anion gap	0.005	6	46	1.045	0.409
Neutrophils	**0.245**	**6**	**52**	**4.145**	**0.002**
Lymphocytes	**0.254**	**6**	**52**	**4.289**	**0.001**

Figures in bold are significantly different at α < 0.05.

**Table 3:** Non-parametric Kruskal-Wallis test of locality and wing-lesion score impact on blood parameters.

Variable	Wing-lesion score				Locality			
	H	df	n	p	H	df	n	p
Haematocrit	2.613	4	58	0.625	0.222	2	58	0.895
Haemoglobin	2.613	4	58	0.625	0.222	2	58	0.895
Eosinophils	0.756	4	59	0.944	5.242	2	59	0.073
Monocytes	1.382	4	59	0.847	3.201	2	59	0.202
Basophils	1.400	4	59	0.844	0.025	2	59	0.988

**Table 4:** Difference between healthy and diseased hibernating bats in groups defined by a) principal component analysis (PCA) and b) UV spot threshold (total UV-fluorescing skin lesion number = 328.5).

*Variables*	*Groups defined by PCA*					*Groups defined by skin lesion threshold*				
	Mean healthy	Mean diseased	t	df	p	Mean healthy	Mean diseased	t	df	p
Na	154.177	151.333	1.521	73	0.133	153.811	151.955	0.961	73	0.340
K	6.62	7.1125	−1.564	73	0.122	6.738	6.873	−0.412	73	0.682
Cl	122.647	124.083	−0.756	73	0.452	122.962	123.455	−0.252	73	0.802
tCO_2_	**24.902**	**22.958**	**2.109**	**73**	**0.038**	24.698	23.273	1.488	73	0.141
Urea	20.492	24.217	−1.903	73	0.061	20.862	23.664	−1.381	73	0.171
Glucose	**7.102**	**4.521**	**5.599**	**73**	**<0.001**	**6.700**	**5.255**	**2.683**	**73**	**0.009**
Haematocrit	55.177	57.083	−1.799	73	0.076	55.717	55.955	−0.214	73	0.831
pH	**7.294**	**7.249**	**3.037**	**73**	**0.003**	7.286	7.262	1.540	73	0.128
pCO_2_	6.415	6.558	−0.646	73	0.52	6.474	6.431	0.190	73	0.850
HCO_3_	**23.477**	**21.471**	**2.215**	**73**	**0.03**	23.223	21.9	1.398	73	0.166
Base excess	**−3.059**	**−5.833**	**2.599**	**73**	**0.011**	−3.453	−5.136	1.495	73	0.139
Anion gap	**14.813**	**13.238**	**2.260**	**67**	**0.027**	14.500	13.895	0.817	67	0.417
Haemoglobin	187.549	194.083	−1.811	73	0.074	189.415	190.182	−0.203	73	0.840
Neutrophils	32.114	38.455	−1.346	55	0.184	31.974	39.737	−1.607	55	0.114
Lymphocytes	67.371	59.864	1.616	55	0.112	67.184	59.053	1.699	55	0.096
Eosinophils	**0.457**	**1.273**	**−2.800**	**55**	**0.007**	0.658	1.000	−1.075	55	0.288
Monocytes	0.286	0.364	−0.319	55	0.751	0.368	0.211	0.628	55	0.533
Basophils	0.114	0.046	0.579	55	0.565	0.132	0.000	1.080	55	0.285
Body surface temperature	**7.906**	**5.933**	**4.326**	**72**	**<0.001**	**7.698**	**6.176**	**3.042**	**72**	**0.003**
Body mass index	**0.401**	**0.382**	**2.338**	**73**	**0.022**	0.397	0.390	0.900	73	0.371
log *P. destructans* load	**−1.102**	**−0.186**	**−3.584**	**71**	**<0.001**	**−1.168**	**0.064**	**−5.033**	**71**	**<0.001**

Figures in bold are significantly different at α < 0.05.

## Discussion

The continent-wide colonisation of Palearctic underground hibernacula and ongoing spread of the fungal pathogen in North America makes exposure of bats hibernating in the temperate zone of the northern hemisphere to *P. destructans* highly probable^11,16,17,20,21,55–58^. The wide distribution of both the host and its pathogen results in spatial and temporal variation in host-pathogen population interactions, allowing performance studies into host health and pathogen virulence under differing environmental conditions. Here we show that even early-stage fungal damage of bat wing membranes may negatively impact physiological status, dependent on infection intensity and hibernation conditions. Our data suggest that the pattern of disease impact can vary between sites.

We chose European *M. myotis* as a model species to examine naturally occurring host-pathogen interactions with *P. destructans* as they have a higher survival capacity than the North American little brown bat (*M. lucifugus*) and big brown bat (*Eptesicus fuscus*)^59^. Nevertheless, sporadic mortalities associated with *P. destructans* infection have been reported for *M. myotis*, documenting that infection intensity can range from mild to severe^18^. Recently, a model taking account of temperature, humidity-dependent fungal growth and bat energetics during hibernation was devised, which predicted that the likelihood of surviving *P. destructans* infection increases with increasing body size and drier and/or colder hibernation sites^59^. While *M. myotis* is one of the largest European bat species, highest infection intensity has been found in those hibernating at low hibernation body temperatures, contrary to our prediction. Three hypotheses may explain this paradox. First, bats select lower hibernation temperatures as an adaptation to conserve energy when dealing with high infection intensity. Second, bats selecting low hibernation temperatures develop increased infection intensities as a consequence of a reduced ability to up-regulate immune functions and clear the infection^24,60,61^. Third, under conditions of natural infection, *P. destructans* growth and virulence is stronger in those bats hibernating at low temperatures, despite laboratory studies suggesting a temperature optimum of between 12.5 and 15.8 °C^62^. Unfortunately, we lack data on hibernation temperature history, arousal frequency, infection dose and duration of infection for each bat, which would allow these hypotheses to be tested explicitly. In the present study, bats were only sampled once at the end of the hibernation period. Interestingly, skin lesion number differed between the left and right wings, suggesting differing exposure to infection and uneven spread of the fungal agent across the body surface during cleaning when aroused. A field experiment analysing fungal load dynamics in relation to wing membrane pathology and bat body temperature during hibernation is needed to provide greater insight into such host-pathogen interactions under natural conditions.

### BMI loss in diseased bats indicative of disease tolerance

Our data provide further evidence for tolerance of Palearctic bat species to the *P. destructans* fungus^21^. The regression lines obtained by plotting host BMI against pathogen burden (skin fungal load) at each hibernaculum display a shallow BMI loss slope as infection intensity increases (Fig. 2). Owing to the differences in origin of the hibernacula used in this study, host-pathogen population interactions will have undergone different evolutionary histories, ranging from tens, to hundreds or even thousands of years. Cave-hibernating bats, with their distinctly lower BMI (Fig. 2), showed lowest tolerance capacity to infection. Transition to euthermia in the early post-hibernation period allows bats to mount an effective immune response against the fungal pathogen and clear any skin infection^19,63^. Later, the bat’s defence strategy turns from tolerance to resistance^42^. Very little is known about the health of *P. destructans* infected hosts in the period following emergence from hibernation. The dichotomy of disease outcome in euthermic bats results in either healing^63^ or mortality due to immunopathology^64^. In *M. myotis*, healing may occur within two weeks, during which the diagnostic UV-fluorescence disappears and a scab develops over the previously infected skin^19^. The costs of neutrophilic inflammation and wing membrane tissue remodelling are hard to estimate. Likewise, we lack detailed quantification of physiological costs associated with flight performance and changes in foraging efficiency in bats recovering from *P. destructans* infection^63,65–67^. Upon arousal, early euthermic females may also face a trade-off between mounting an immune response and the energetic investment needed to initiate gestation^68,69^. Higher cortisol levels, indicative of chronic stress, have been recorded in bats surviving exposure to *P. destructans*, and this may have adverse effects on reproductive success^70^. Further, North American species recovering from *P. destructans* infection have shown shifts in pregnancy and lactation, suggestive of reproductive fitness consequences^71^. Similar studies on reproductive fitness consequences have yet to be performed on European bats facing fungal pathogen pressure.

### Alterations in blood homeostasis in diseased bats

While infectious diseases are commonly thought to induce biochemical responses that differ between species^72^, our data showed hibernation site-specific differences. Haematology and blood chemistry reflect body and tissue status. A range of mechanisms maintain blood parameters within a narrow range; blood pH, for example, being maintained through respiratory system and kidney function. Hibernation, on the other hand, represents a specific physiological state resulting in changes to metabolic and biochemical pathways^73^. Heart rate, cardiac output and respiration are greatly reduced  during deep hibernation, and these changes lead to a drop in pH and marked acidosis.

Studies of blood homeostasis in *M. lucifugus* indicate a pattern of changes dependent on WNS intensity^29,31,32,74^. While such studies have sampled blood by decapitation, we used non-lethal vessel puncturing to study *M. myotis*, a strictly protected European bat. Cryan *et al*.^31^, using data on *P. destructans* infection in both captive and wild hibernating bats, noted that electrolyte depletion increased with increasing wing damage severity. Furthermore, measurements of urine-specific gravity suggested that bats underwent hypotonic dehydration. In a second study, captive hibernation of *M. lucifugus* following experimental inoculation with *P. destructans* complicated blood sampling, allowing analysis of only eight infected bats^32^. The addition of data from two follow-up captive inoculation experiments, however, showed no difference between the infected and control groups^68^. While data obtained by Warnecke *et al*.^32^ were only suggestive of metabolic acidosis, Verant *et al*.^29^ observed chronic respiratory acidosis with metabolic compensation in bats at an early stage of the disease.

### A skin lesion threshold distinguishing healthy and diseased bats

As all bats in our study were naturally infected in their hibernacula and confirmed positive for *P. destructans* (with the exception of one individual from Nietoperek), it was not possible to compare host physiological responses to the fungus against a non-infected control group. Nevertheless, our non-diseased and diseased groups, as defined by PCA, differed in blood pH, tCO_2_, bicarbonate, base excess/deficit and anion gap. These acid-base parameters shifted to mild metabolic acidosis in the diseased group (Table 4). The diseased group displayed higher infection intensity, distinguished by both fungal load and UV-fluorescing skin lesions. Bats defined as diseased (i.e. with blood parameters showing homeostasis disruption) had a hibernating body surface temperature around 2 °C lower than non-diseased individuals. The diseased group also displayed significantly decreased glucose concentrations and BMI. Contrary to blood biochemistry results for *M. lucifugus*, we observed no differences in electrolytes between diseased and non-diseased *M. myotis*, suggesting that the acid-base disruption was due to increased energy utilisation associated with infection. The increase in differential neutrophil count was non-significant, probably because the white blood cells migrated from blood to the infected sites^19^. Interestingly, significant eosinophilia was observed in diseased *M. myotis*. Peripheral blood eosinophilia is commonly associated with chronic, parasitic and fungal infections^75^. As eosinophilia is also associated with hypersensitivity reactions, however, our findings may support the hypothesis that immunopathology plays a role in post-emergent WNS mortality^64^.

While exposure of bats to multiple natural and/or anthropogenic stressors is a realistic environmental scenario^76–79^, sub-lethal adverse effects are mostly underreported. Disease pathogenesis and the action of multiple stressors during hibernation are not yet fully understood; however, different stressors may well combine to exert synergistic effects^80^. Importantly, disturbance by human activities, such as tourism, caving or research, could also threaten hibernating bats by increasing energy expenditure^81^.

## Conclusion

Following the emergence of WNS and recognition of its impact on North American bat populations in 2006, chiropterologists concerned with European bat conservation have asked one essential question: are Palearctic bat populations and communities threatened by this fungal disease? Up to now, there have been no functional studies addressing host-pathogen interactions in relation to WNS. However, there is mounting evidence for virulent skin invasion and pathognomonic lesions in many hibernating Eurasian bat species. As these findings have not been associated with mass mortalities and/or population declines, research should be directed toward examining health consequences in terms of trade-off mechanisms modulating investment into host response to infection.

In this study, we were able to show variation in fungal pathogen pressure in relation to hibernaculum-dependent physiological effects of *P. destructans* infection. We conclude that European *M. myotis* survive *P. destructans* invasion, despite showing deterioration in health, with infection intensity dependent on hibernation conditions. Disruption in blood homeostasis was observed in bats, even with a low threshold number of skin lesions on both wings. We argue that overwintering in underground hibernacula colonised by this virulent pathogen is associated with health-related costs for European bats. Further research should aim to quantify levels of homeostasis disruption in terms of constrained energy reserves and compatibility for survival.

## Electronic supplementary material

Supplementary Table S1
