# Supplementary material for: Alterations in the health of hibernating bats under pathogen pressure
Source: Sci Rep. 2018 Apr 17;8:6067. doi: 10.1038/s41598-018-24461-5 (PMC5904171; doi:10.1038/s41598-018-24461-5)
Supplement: Supplementary file 1 — Supplementary Table S1 [file 41598_2018_24461_MOESM1_ESM.pdf]

Title: Alterations in the health of hibernating bats under pathogen pressure

Authors: Hana Bandouchova, Tomáš Bartonička, Hana Berkova, Jiri Brichta, Tomasz Kokurewicz, Veronika Kovacova, Petr Linhart, Vladimir Piacek, Jiri Pikula, Alexandra Zahradníková Jr., Jan Zukal

Supplementary Table S1: Data on fungal load, number of ultra-violet fluorescent skin lesions and blood parameters in *Myotis myotis* bats from Nietoperek (Poland), the Šimon and Juda mines (Czech Republic) and the Sloupsko-šošůvské caves (Czech Republic)

Country	Locality	Bat	Na	K	Cl	tCO2
Poland	Nietoperek	PLMMyo1	159	6.5	119	32
Poland	Nietoperek	PLMMyo2	155	7.9	121	27
Poland	Nietoperek	PLMMyo3	152	5	116	27
Poland	Nietoperek	PLMMyo4	147	8.3	119	25
Poland	Nietoperek	PLMMyo5	150	7.3	117	29
Poland	Nietoperek	PLMMyo6	158	5.6	124	23
Poland	Nietoperek	PLMMyo7	148	6.4	111	33
Poland	Nietoperek	PLMMyo8	160	5.9	130	30
Poland	Nietoperek	PLMMyo9	148	6	117	21
Poland	Nietoperek	PLMMyo10	155	8.2	118	31
Poland	Nietoperek	PLMMyo11	168	5.3	128	31
Poland	Nietoperek	PLMMyo12	171	5.3	137	23
Poland	Nietoperek	PLMMyo13	168	7	127	29
Poland	Nietoperek	PLMMyo14	168	8.2	139	20
Poland	Nietoperek	PLMMyo15	161	8	135	23
Poland	Nietoperek	PLMMyo17	156	8.1	120	26
Poland	Nietoperek	PLMMyo18	156	5.9	117	33
Poland	Nietoperek	PLMMyo19	149	5.6	114	29
Poland	Nietoperek	PLMMyo20	156	4.8	119	29
Poland	Nietoperek	PLMMyo21	152	8.2	115	28
Poland	Nietoperek	PLMMyo22	124	6.2		22
Czech Republic	Mala Moravka	MMMyo1	151	5.6	115	29
Czech Republic	Mala Moravka	MMMyo2	149	6	118	22
Czech Republic	Mala Moravka	MMMyo3	152	6.5	120	24
Czech Republic	Mala Moravka	MMMyo4	152	9.1	129	22
Czech Republic	Mala Moravka	MMMyo5	161	6.3	135	22
Czech Republic	Mala Moravka	MMMyo6	162	7.7		
Czech Republic	Mala Moravka	MMMyo7	148	8.1	117	26
Czech Republic	Mala Moravka	MMMyo8	151	4.3	119	24
Czech Republic	Mala Moravka	MMMyo9	149	6.7	118	25
Czech Republic	Mala Moravka	MMMyo10	149	7.7	124	18
Czech Republic	Mala Moravka	MMMyo11	157	7.7	136	18
Czech Republic	Mala Moravka	MMMyo12	143	7.1	109	26
Czech Republic	Mala Moravka	MMMyo13	152	6.5	119	23
Czech Republic	Mala Moravka	MMMyo14	143	8.2	115	22
Czech Republic	Mala Moravka	MMMyo16	141	6.4	119	17
Czech Republic	Mala Moravka	MMMyo17	146	8.5	122	21
Czech Republic	Mala Moravka	MMMyo18	138	9.1	113	23
Czech Republic	Mala Moravka	MMMyo19	134	6.1	114	16
Czech Republic	Mala Moravka	MMMyo20	133	8.5	103	21
Czech Republic	Mala Moravka	MMMyo30	142	7.7	115	17
Czech Republic	Mala Moravka	MMMyo31	145	9.1	130	22
Czech Republic	Sloupsko-šošůvské caves	SLMMyo1	151	7.4	124	25
Czech Republic	Sloupsko-šošůvské caves	SLMMyo2	152	5.7	115	27
Czech Republic	Sloupsko-šošůvské caves	SLMMyo4	159	6.3	133	21
Czech Republic	Sloupsko-šošůvské caves	SLMMyo5	157	6.5	125	27
Czech Republic	Sloupsko-šošůvské caves	SLMMyo6	159	5.6	130	20
Czech Republic	Sloupsko-šošůvské caves	SLMMyo7	158	8.5	127	26
Czech Republic	Sloupsko-šošůvské caves	SLMMyo8	155	8	127	21

Czech Republic	Sloupsko-šošůvské caves	SLMMyo9	156	6.3	125	24
Czech Republic	Sloupsko-šošůvské caves	SLMMyo10	156	5.8	126	21
Czech Republic	Sloupsko-šošůvské caves	SLMMyo11	150	6	119	21
Czech Republic	Sloupsko-šošůvské caves	SLMMyo12	159	5.7	131	18
Czech Republic	Sloupsko-šošůvské caves	SLMMyo13	151	4.9	120	27
Czech Republic	Sloupsko-šošůvské caves	SLMMyo15	157	6.8	126	23
Czech Republic	Sloupsko-šošůvské caves	SLMMyo16	145	7.8	123	22
Czech Republic	Sloupsko-šošůvské caves	SLMMyo17	157	7.3	132	24
Czech Republic	Sloupsko-šošůvské caves	SLMMyo18	160	7.7	128	27
Czech Republic	Sloupsko-šošůvské caves	SLMMyo19	146	7.2	122	22
Poland	Nietoperek	PL1	163	5.9	132	24
Poland	Nietoperek	PL2	158	5.1	125	25
Poland	Nietoperek	PL3	142	6.5	111	28
Poland	Nietoperek	PL4	159	6.3	131	24
Poland	Nietoperek	PL5	161	6	132	23
Poland	Nietoperek	PL6	158	5.9	133	21
Poland	Nietoperek	PL7	147	8.5	121	25
Poland	Nietoperek	PL8	164	5.7	130	
Poland	Nietoperek	PL9	157	5.8	129	25
Poland	Nietoperek	PL10	162	9.1	136	23
Poland	Nietoperek	PL11	162	7.3	136	20
Poland	Nietoperek	PL12	151	9.1	130	21
Poland	Nietoperek	PL13	145	6.1	117	24
Poland	Nietoperek	PL14	167	6.6	131	26
Poland	Nietoperek	PL15	151	3.2	117	27
Poland	Nietoperek	PL16	156	6.6	124	28
Poland	Nietoperek	PL18	152	7.3	127	21
Poland	Nietoperek	PL19	152	6	121	26
Poland	Nietoperek	PL20	151	4.8	114	27
Poland	Nietoperek	PL21	156	4.5	124	

Urea	Glucose	Haematocrit	pH	pCO2	HCO3	BE	AnGAP	Hb
27.6	10	60	7.263	8.95	30.3	3	16	204
19	7.7	58	7.279	7.32	25.7	-1	17	197
6	5.7	49	7.382	5.7	25.4	0	15	167
14	7.1	59	7.275	6.73	23.5	-3	13	201
24.3	5.9	59	7.363	6.5	27.7	2	13	201
18.5	7.5	54	7.288	6.15	22.1	-5	18	184
9	4.4	56	7.365	7.31	31.3	6	12	190
20.3	1.5	55	7.303	7.48	27.8	1	8	187
10.1	7.7	54	7.3	5.37	19.8	-7	17	184
36	6.3	55	7.3	7.94	29.3	3	15	187
17.6	5.9	53	7.343	7.28	29.7	4	15	180
28.7	5.6	60	7.281	6.07	21.4	-5	18	204
19.7	6.7	61	7.275	7.94	27.7	1	20	207
27.3	8	55	7.258	5.51	18.5	-9	19	187
38.7	4.9	57	7.298	5.82	21.4	-5	13	194
27.3	4.9	61	7.378	5.58	24.7	0	20	207
20.7	5.7	54	7.384	7.07	31.7	7	13	184
13	8.4	56	7.433	5.46	27.4	3	13	190
23.2	5.5	58	7.362	6.45	27.4	2	14	197
10.3	5.9	55	7.308	7.13	26.8	1	18	187
33	11.6	39	7.332	5.17	20.6	-5		133
16	4.2	57	7.371	6.44	28	3	13	194
20.5	6.8	55	7.187	7.07	20.1	-8	17	187
20.1	7.2	56	7.346	5.64	23.2	-3	15	190
25.2	3.1	65	7.225	6.59	20.5	-7		221
27	2.7	60	7.232	6.61	20.9	-7	11	204
11.9	5.4	51	7.268	7.2	24.6	-2	14	173
15.2	5.4	56	7.262	6.64	22.5	-5	13	190
16.6	5.7	58	7.281	6.69	23.6	-3	14	197
24.9	7	53	7.244	5.2	16.9	-10	16	180
35.7	3.4	55	7.12	6.81	16.6	-13	12	187
10	7.2	54	7.244	7.48	24.2	-3	17	184
21.7	6.9	57	7.291	5.98	21.6	-5	18	194
15.7	8.8	56	7.289	5.67	20.4	-6	16	190
17.7	12	47	7.222	5.09	15.7	-12	13	160
24.6	5.2	55	7.256	5.77	19.3	-8	12	187
19.9	9.8	57	7.301	5.91	21.9	-5		194
48.9	5.8	64	7.223	4.82	14.9	-13		218
17.6	6.5	57	7.383	4.49	20.1	-5	18	194
31.2	9.4	57	7.137	6.29	16	-13	18	194
31.7	4.3	58	7.079	8.91	19.8	-10		197
26.4	4.7	54	7.225	7.48	23.2	-4	11	184
13.2	5.8	55	7.262	7.5	25.4	-2	17	187
32.5	6.6	57	7.256	5.98	19.9	-7	13	194
24.1	8.2	58	7.237	7.95	25.4	-2	13	197
18.9	4.4	54	7.179	6.49	18.1	-10	16	184
29.8	3.4	62	7.332	6.12	24.3	-2	15	211
35	6	59	7.181	6.86	19.3	-9	17	201

21.4	4.2	62	7.299	6.06	22.3	-4	15	211
16.9	7.3	45	7.225	6.4	19.9	-8	15	153
11.8	7.8	54	7.245	5.93	19.3	-8	17	184
27.5	7.1	57	7.235	5.2	16.5	-11	17	194
11.5	4.8	50	7.276	7.29	25.4	-1	10	170
20.9	5.2	61	7.278	6.22	21.9	-5	16	207
21.2	4.2	54	7.295	5.68	20.7	-6	10	184
40.5	2.7	51	7.296	6.19	22.7	-4	9	173
21.9	4.1	63	7.211	8.36	25.2	-3	15	214
15.5	3.8	51	7.264	5.96	20.2	-7	11	173
19	5	58	7.316	5.9	22.6	-4	14	197
23.6	9.4	56	7.294	6.41	23.3	-3	14	190
14.3	6.1	46	7.397	5.84	27	2	10	156
22.2	5.6	56	7.275	6.51	22.7	-4	12	190
16.6	6.4	58	7.265	6.46	22	-5	14	197
33	5.6	53	7.243	6.01	19,4	-8	12	180
17.3	6.5	46	7.264	6.83	23.2	-4	11	156
40.5	6.5	57						194
23.3	4.9	58	7.286	6.69	23.9	-3	10	197
22.3	6.4	66	7.226	6.82	21.3	-6		224
32.3	14.8	52	7.289	5.19	18.7	-8	15	177
20.7	10.1	55	7.282	5.55	19.7	-7		187
19.2	7.5	55	7.341	5.67	23	-3	12	187
18.1	10.3	61	7.249	7.34	24.1	-3	18	207
12.9	7.2	53	7.29	7.12	25.7	-1	12	180
19.6	8	56	7.342	6.46	26.3	1	13	190
27.3	2.8	57	7.257	5.96	19.9	-7	12	194
19.8	6.7	48	7.283	6.78	24.1	-3	13	163
12.4	5	46	7.341	6.3	25.6	0	16	156
15.5	8.8	51						173

Neutrophils	Lymphocytes	Eosinophils	Monocytes	Basophils	Skin lesions (UV left wing)
9	88	1	2	0	13
8	90	1	1	0	6
16	81	0	0	3	10
17	82	2	1	0	129
43	56	0	1	0	30
14	85	0	1	0	5
7	93	0	0	0	0
13	87	0	0	0	52
14	86	0	0	0	25
13	85	1	1	0	13
36	64	0	0	0	38
20	80	0	0	0	34
23	77	0	0	0	3
13	87	0	0	0	50
76	23	0	1	0	125
39	61	0	0	0	0
32	68	0	0	0	87
57	42	1	0	0	5
28	72	0	0	0	6
25	75	0	0	0	116
30	69	1	0	0	72
55	45	0	0	0	681
41	57	2	0	0	168
54	45	0	1	0	283
51	46	3	0	0	696
42	57	1	0	0	67
83	17	0	0	0	3013
30	68	1	1	0	521
22	76	1	1	0	268
42	56	2	0	0	114
52	48	0	0	0	682
75	25	0	0	0	860
40	59	1	0	0	85
56	44	0	0	0	24
39	59	1	0	1	194
70	30	0	0	0	126
48	52	0	0	0	214
21	89	0	0	0	123
43	57	0	0	0	432
46	53	1	0	0	83
60	40	0	0	0	34
27	68	5	0	0	860
28	71	1	0	0	116
19	74	0	6	1	24
20	79	0	1	0	133
37	63	0	0	0	9
20	77	3	0	0	106
45	55	0	0	0	72
34	62	4	0	0	91

43	57	0	0	0	193
36	62	2	0	0	192
8	89	3	0	0	0
32	67	1	0	0	69
39	61	0	0	0	103
44	54	2	0	0	211
51	47	2	0	0	60
15	84	1	0	0	295
55	45	0	0	0	26
27	72	1	0	0	13
					249
					0
					8
					2
					0
					0
					3
					5
					2
					1
					2
					36
					4
					4
					29
					27
					302
					421
					125
					3

Skin lesions (UV right wing)	Skin lesions (UV total)	Hibernation temperature
13	26	7.6
1	7	8.6
2	12	8.6
35	164	9.3
89	119	9.8
1	6	11
2	2	8.3
8	60	8.5
114	139	8.5
16	29	8.6
244	282	8.1
29	63	7.6
19	22	7.8
69	119	8.4
96	221	8.7
0	0	10.8
476	563	9.5
21	26	9.4
10	16	9.3
241	357	
60	132	
1234	1915	4.6
337	505	5.2
236	519	5.3
374	1070	4.5
86	153	4.2
3782	6795	4.6
685	1206	4.3
512	780	4.5
242	356	5.2
132	814	4.6
1534	2394	5.3
84	169	4.5
24	48	4.3
102	296	4.6
104	230	4.6
176	390	4.2
157	280	4.6
444	876	5.3
46	129	4.2
148	182	5.2
630	1490	4.3
148	264	6.1
56	80	6.2
283	416	6.3
14	23	6.3
58	164	6.4
96	168	6.6
93	184	6.4

413	606	6.7
237	429	6.5
2	2	6.3
173	242	6.5
210	313	6.5
358	569	6.3
49	109	6.3
310	605	6.4
49	75	6.4
3	16	6.4
220	469	9.3
0	0	9.9
10	18	10.7
5	7	10.1
2	2	9.3
7	7	11.1
35	38	8.7
8	13	8.9
1	3	8.7
0	1	8.6
27	29	8.5
45	81	8.4
4	8	8.4
17	21	8
104	133	8.5
8	35	8.6
348	650	11
34	455	10.4
12	137	9
19	22	8.7

Forearm length	Body mass	BMI	Date of sampling	Sex
63.3	29.5	0.466034755134281	19.03.2015	f
62.8	22.5	0.35828025477707	19.03.2015	m
62.2	27	0.434083601286174	19.03.2015	f
61	26	0.426229508196721	19.03.2015	f
59.4	23	0.387205387205387	19.03.2015	m
58.9	23.5	0.398981324278438	19.03.2015	m
60.6	26.5	0.437293729372937	19.03.2015	m
57.9	22	0.379965457685665	19.03.2015	m
58.4	22.5	0.38527397260274	19.03.2015	f
63.2	26	0.411392405063291	19.03.2015	f
61.7	24.5	0.39708265802269	19.03.2015	f
59.1	21.5	0.363790186125212	19.03.2015	m
62.4	27	0.432692307692308	19.03.2015	f
60.2	23	0.382059800664452	19.03.2015	f
60.3	23.5	0.38971807628524	19.03.2015	f
60.9	22.5	0.369458128078818	19.03.2015	m
60	24	0.4	19.03.2015	m
60.9	25.5	0.41871921182266	19.03.2015	m
59.7	24	0.402010050251256	19.03.2015	m
60.1	22	0.366056572379368	19.03.2015	f
57.7	21.5	0.37261698440208	19.03.2015	m
59.2	22.8	0.385135135135135	03.04.2015	m
60.2	26	0.431893687707641	03.04.2015	m
59.6	23.2	0.389261744966443	03.04.2015	m
60.8	27.2	0.447368421052632	03.04.2015	m
58.3	24.8	0.425385934819897	03.04.2015	m
65.1	23.4	0.359447004608295	03.04.2015	f
61.3	25.2	0.411092985318108	03.04.2015	f
61.2	27.5	0.449346405228758	03.04.2015	m
61.4	25.5	0.415309446254072	03.04.2015	m
59	22.5	0.38135593220339	03.04.2015	m
59.3	22	0.370994940978078	03.04.2015	m
62.2	24.6	0.395498392282958	03.04.2015	m
58.8	24.8	0.421768707482993	03.04.2015	m
62	24.5	0.395161290322581	03.04.2015	f
59.7	24.2	0.40536013400335	03.04.2015	m
60.2	24	0.398671096345515	03.04.2015	m
60.6	21.8	0.35973597359736	03.04.2015	m
60.4	23.6	0.390728476821192	03.04.2015	m
64.2	25.5	0.397196261682243	03.04.2015	f
60.4	23.5	0.389072847682119	03.04.2015	m
60.7	23.5	0.387149917627677	03.04.2015	m
61.2	19.5	0.318627450980392	17.04.2015	f
59.8	23	0.384615384615385	17.04.2015	m
60	21.5	0.358333333333333	17.04.2015	m
57.7	21	0.363951473136915	17.04.2015	m
61.9	22.5	0.363489499192246	17.04.2015	f
58.2	21.5	0.369415807560137	17.04.2015	m
61.8	21.5	0.34789644012945	17.04.2015	f

59.7	22	0.368509212730318	17.04.2015	m
58.6	19	0.324232081911263	17.04.2015	m
60.6	24	0.396039603960396	17.04.2015	m
59.5	21	0.352941176470588	17.04.2015	m
62.8	21.5	0.342356687898089	17.04.2015	f
58.1	22.5	0.387263339070568	17.04.2015	m
60.4	23	0.380794701986755	17.04.2015	m
61	19	0.311475409836066	17.04.2015	m
62.2	23	0.369774919614148	17.04.2015	f
61.9	23	0.37156704361874	17.04.2015	f
65.3	25.5	0.390505359877489	19.03.2016	f
61.1	27	0.44189852700491	19.03.2016	f
59.8	23.5	0.392976588628763	19.03.2016	m
62.2	27	0.434083601286174	19.03.2016	f
58.9	26.5	0.449915110356537	19.03.2016	f
62.3	24.5	0.393258426966292	19.03.2016	f
63.4	29.5	0.465299684542587	19.03.2016	f
62.7	24.5	0.390749601275917	19.03.2016	f
64.7	26	0.401854714064915	19.03.2016	f
61.6	23.5	0.381493506493506	19.03.2016	f
62.3	23.5	0.377207062600321	19.03.2016	f
60.8	24.5	0.402960526315789	19.03.2016	f
63.7	26	0.408163265306122	20.03.2016	f
62.2	27.5	0.442122186495177	20.03.2016	f
61	26.5	0.434426229508197	20.03.2016	m
61	23.5	0.385245901639344	20.03.2016	f
61.4	22.5	0.366449511400651	20.03.2016	f
63.2	28	0.443037974683544	20.03.2016	f
63.4	29	0.457413249211356	20.03.2016	f
64.4	26.5	0.411490683229814	20.03.2016	f

Age	*P. destructans* load
ad	0.0544381576671936
sad	0.0195816132423898
ad	0.0315364732510184
ad	0.406571263383282
ad	0.222597436516891
ad	0.0408112831287848
sad	0
ad	0.0233714060970526
ad	0.00549888258219571
ad	0.113760301053183
ad	0.059747049689906
ad	0.0675026806788231
ad	0.041553840143751
ad	0.431682949720439
ad	0.0144964924865044
ad	0.0537394386314814
sad	0.121088705876972
ad	0.0320096283950525
sad	0.210114529801154
ad	2.1192557618197
ad	
ad	0.293016297584994
sad	2.15575309740245
ad	5.89379001335643
ad	6.28658041074651
sad	0.116562630739205
ad	12.8274274255086
ad	1.98338138425553
ad	3.453464712462
ad	1.18362826207467
ad	7.51256395713623
ad	3.18607350575418
ad	0.254616524301805
ad	0.214000948045937
ad	7.50438012445076
ad	3.53494002926166
ad	2.80108679569021
sad	0.444932395635932
ad	4.90667606981103
ad	0.489811963348835
ad	
ad	
ad	0.0379206345668535
ad	0.646759896423162
ad	0.268637359142226
ad	0.00807725522405844
ad	1.9553719533215
ad	0.57364042054793
sad	1.27785721871048

ad	0.158241916070859
sad	2.2474165598117
sad	0.128478183413083
sad	1.44281710450792
ad	0.379148757898925
ad	0.463752372086016
ad	0.107421748315307
sad	6.31107380153283
sad	0.250009532607537
ad	2.89837298388187
ad	0.431108757145475
ad	0.00186974011587723
ad	0.00560856275655036
ad	0.00688468798755705
ad	0.00102746008112895
ad	0.00105081983519282
ad	0.00169423861177047
ad	0.0243818850467132
ad	0.000546370253145353
ad	0.00611631201645431
sad	0.113085104867628
ad	0.0982746697209541
ad	0.0033280176280996
ad	0.139322409362806
ad	0.212304323216677
ad	0.0134815948574919
ad	1.58324203414883
ad	0.00722672012886443
ad	0.000726740061844778
ad	0.00326344712294369
